# Does Preliminary Chest Shape Assessment Improve the Prognostic Risk Stratification of Symptomatic Individuals with Primary Mitral Regurgitation? A Narrative Review of Traditional and Innovative Prognostic Indicators

**DOI:** 10.3390/jcm14238297

**Published:** 2025-11-22

**Authors:** Andrea Sonaglioni, Gian Luigi Nicolosi, Michele Lombardo, Massimo Baravelli

**Affiliations:** 1 Division of Cardiology, IRCCS MultiMedica, 20123 Milan, Italy; michele.lombardo@multimedica.it (M.L.); massimo.baravelli@multimedica.it (M.B.); 2 Division of Cardiology, Policlinico San Giorgio, 33170 Pordenone, Italy; gianluigi.nicolosi@gmail.com

**Keywords:** primary mitral regurgitation, prognostic risk stratification, myocardial strain parameters, mitral annular disjunction, exercise stress echocardiography

## Abstract

Primary mitral regurgitation (PMR) is the most common mitral valve disorder in clinical practice. Although several prognostic indicators derived from resting transthoracic echocardiography (TTE) and exercise stress echocardiography (ESE) are available, patient outcomes remain highly variable, with substantial differences in complication rates and mortality. Identifying individuals at lower cardiovascular risk is therefore clinically relevant, as they represent a large proportion of cases. Current guidelines recommend an integrative approach—combining qualitative, semi-quantitative, and quantitative indices—to determine the timing of intervention, but they do not specifically address risk stratification in low-risk PMR populations. Recent studies have highlighted the potential prognostic value of chest wall configuration, assessed noninvasively using the Modified Haller Index (MHI). Defined as the ratio of latero-lateral thoracic diameter to the antero-posterior (A-P) sternum–spine distance, MHI appears to influence myocardial deformation indices obtained by speckle-tracking echocardiography (STE). Patients with PMR due to mitral valve prolapse (MVP) often show a reduced A-P thoracic diameter caused by sternal depression. Among these, those with an MHI > 2.5 or A-P diameter ≤ 13.5 cm display greater impairment in global and basal strain, particularly in longitudinal and circumferential directions. These abnormalities likely reflect extrinsic geometric constraints and cardiac displacement leading to apparent dyssynchrony rather than intrinsic myocardial dysfunction. A reduced A-P diameter was also independently associated with mitral annular disjunction (MAD) in MVP and emerged as a determinant of impaired strain in this subgroup. In a retrospective cohort of 424 symptomatic MVP patients with moderate MR undergoing ESE, positive tests and exercise-induced severe MR were uncommon. Importantly, an MHI > 2.5 or an A-P diameter ≤ 13.5 cm was associated with a favorable medium-term prognosis, with few adverse cardiovascular events. This narrative, non-systematic review, based on a structured but non-PRISMA literature search, summarizes current evidence on conventional and novel echocardiographic prognostic markers and their implications for risk stratification in PMR. As such, it carries inherent limitations, including potential selection bias, incomplete retrieval of unpublished or negative studies, and reliance on single-center observational data. The findings should therefore be interpreted cautiously and validated through larger, independent, multicenter investigations.

## 1. Introduction

Mitral regurgitation (MR) is the second most prevalent valvular heart condition in Europe, surpassed only by aortic stenosis, with primary mitral regurgitation (PMR) accounting for approximately 61–67% of cases [1,2]. PMR is characterized by systolic regurgitant flow from the left ventricle (LV) to the left atrium (LA), arising from intrinsic abnormalities of the mitral valve (MV) apparatus, including the leaflets, chordae tendineae, or papillary muscles. Structural lesions affecting one or more of these components define PMR. In Western countries, degenerative etiologies, particularly fibroelastic deficiency and Barlow disease, represent the predominant causes [1,3,4].

Chronic significant PMR frequently induces progressive LV remodeling and dysfunction, ultimately predisposing to heart failure and other adverse cardiac outcomes [5]. This disease course often necessitates repeated clinical evaluations, hospital admissions, and surgical intervention [6]. With the aging of the general population, the burden of PMR is anticipated to increase further [7]. Determining the optimal timing for surgical intervention has been the subject of longstanding debate. Current practice increasingly supports earlier surgical referral, as earlier intervention is associated with superior postoperative outcomes. For instance, surgery is now recommended at a left ventricular end-systolic diameter (LVESD) threshold of 40 mm, rather than waiting until it reaches 45 mm [8]. According to current guidelines, the presence of symptoms or LV dysfunction constitutes a Class I indication for surgery in patients with PMR [8,9]. However, reliance on left ventricular ejection fraction (LVEF) and LVESD has limitations, as these indices are influenced by LV geometry, loading conditions, and heart rate [10]. Consequently, these measures may overestimate systolic function in PMR, highlighting the need for more sensitive and reliable markers to guide surgical timing.

Despite numerous prognostic markers evaluated through resting transthoracic echocardiography (TTE) and exercise stress echocardiography (ESE), outcomes in PMR remain heterogeneous, with wide variability in reported rates of complications and mortality [11,12,13,14]. Identifying patients at lower risk is therefore as important as recognizing those at higher risk.

Recent work from our group has highlighted chest wall morphology, quantified by the Modified Haller Index (MHI), as a potential modifier of cardiac function [15]. Multiple studies have shown that an increased MHI (>2.5) influences myocardial strain across different populations—including healthy infants, adults with pectus excavatum, obesity, pregnancy, and individuals with mitral valve prolapse—even in those with preserved systolic function [15,16,17,18,19,20,21]. In symptomatic MVP patients with moderate MR, an MHI ≥2.7 was further associated with excellent prognosis following exercise stress echocardiography [22].

Based on these findings, we hypothesize that incorporating chest wall assessment with MHI into clinical practice may help distinguish compressive causes of symptoms from true pathological dysfunction in symptomatic PMR patients and further enable the identification of a subgroup with a more favorable prognosis. The present narrative review summarizes available evidence on conventional and novel echocardiographic prognostic markers, with emphasis on their threshold values, to refine risk stratification and inform clinical decision-making in PMR.

## 2. Literature Search and Evidence Selection

To provide a comprehensive overview of conventional and emerging prognostic indicators in PMR, we conducted a narrative literature search across PubMed, Scopus, and EMBASE databases, covering publications available up to June 2025. The search strategy combined keywords and MeSH terms including “primary mitral regurgitation,” “echocardiography,” “strain,” “exercise stress echocardiography,” “cardiac magnetic resonance,” “computed tomography,” “nuclear imaging,” “anthropometrics,” “modified Haller index,” and “prognosis.” No language or time restrictions were applied to maximize inclusiveness. Eligible works included observational cohorts, multicenter registries, meta-analyses, and systematic reviews reporting clinical or prognostic outcomes in PMR. Single-center or exploratory studies were considered for their hypothesis-generating value, provided methodological details and outcome data were available. Conference abstracts, case reports, and purely technical or surgical papers without prognostic endpoints were excluded. Reference lists of key publications were manually screened to identify additional relevant studies. This approach aimed to first summarize well-established echocardiographic parameters and then expand to innovative predictors such as strain imaging, exercise stress echocardiography, and anthropometric indices like the MHI.

Because this review was narrative rather than systematic, it does not adhere to PRISMA standards, and potential selection bias cannot be excluded. The absence of formal quality assessment, data extraction forms, and risk-of-bias grading represents a methodological limitation. Nevertheless, efforts were made to ensure balance by integrating independent multicenter data and referencing large guideline-based registries whenever possible.

## 3. Traditional Prognostic Indicators Assessed Using Transthoracic Echocardiography

### 3.1. Echocardiographic Criteria of PMR Severity Recommended by Current Guidelines

Echocardiography remains the cornerstone imaging modality for grading PMR. Current recommendations emphasize an integrative approach that combines qualitative, semiquantitative, and quantitative parameters, together with assessment of LV and LA size [23,24]. Identifying the precise anatomical lesion responsible for MR has clear prognostic significance [4,25] and is critical in determining the feasibility of surgical or transcatheter repair [26,27]. Beyond guidelines, multicenter outcome studies such as the MIDA registry have demonstrated the prognostic importance of echocardiographic grading in degenerative MR with flail leaflet, leading to the development of the MIDA Mortality Risk Score for long-term survival prediction [28].

The main guideline-endorsed echocardiographic indicators of PMR severity [8] are summarized in Table 1.

**Figure 1:**
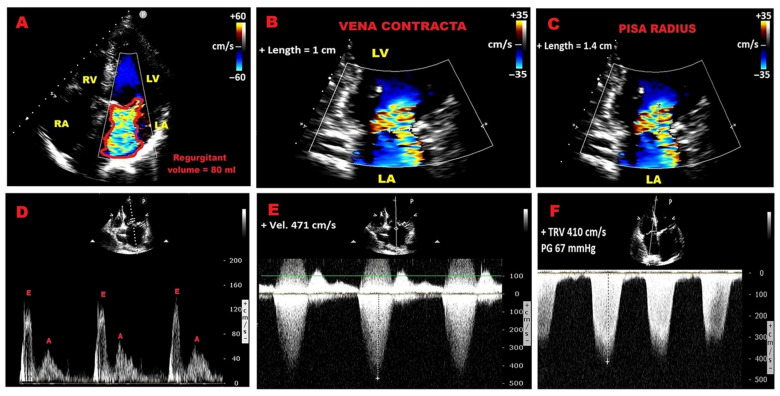
Echocardiographic predictors of adverse outcomes in primary mitral regurgitation, derived from color Doppler, PW–Doppler and CW–Doppler. (**A**) Regurgitant volume. (**B**) Vena contracta width. (**C**) PISA radius. (**D**) Dominant transmitral E wave. (**E**) Dense holosystolic continuous wave Doppler MR jet. (**F**) Increased tricuspid regurgitation velocity. CW, continuous wave; PW, pulsed wave; MR, mitral regurgitation; PISA, proximal isovelocity surface area.

Figure 1 depicts the main traditional parameters derived from color Doppler, pulsed wave (PW) Doppler and continuous wave (CW) Doppler, independently associated with adverse cardiovascular events (negative prognostic indicators) in PMR individuals.

### 3.2. Left Ventricular Size

Among quantitative indices, left ventricular end-systolic diameter (LVESD) is one of the most established prognostic markers in PMR. Longitudinal studies have shown its reproducibility and predictive value for postoperative LV dysfunction [29,30], cardiac events [31], and both early and late mortality [32,33]. Evidence from multicenter registries has confirmed that LVESD independently predicts long-term survival [28]. Thresholds of LVESD ≥ 40 mm and LVEDD > 60 mm have been associated with adverse outcomes, including recurrent MR [33,34,35].

### 3.3. Left Ventricular Function

Left ventricular ejection fraction (LVEF) remains a classical and robust predictor of outcome. Multiple studies have demonstrated its prognostic significance for postoperative dysfunction [36,37,38,39,40], recurrent MR [33], and mortality [33,41]. Although widely used in practice, LVEF abnormalities typically appear late in disease, underscoring the need for complementary imaging parameters in early stratification.

### 3.4. Left Ventricular Global Longitudinal Strain

Most studies consistently show that preoperative impairment in left ventricular global longitudinal strain (LV–GLS) is a reliable prognostic indicator in PMR [42,43,44,45,46]. LV–GLS values between 17.9% and −20.5% are generally considered the thresholds associated with worse postoperative outcomes. Importantly, LV–GLS has been demonstrated to provide incremental prognostic value beyond conventional indices [45,47,48]. In this regard, a recent meta-analysis pooling over 2000 patients confirmed that impaired LV–GLS offers incremental prognostic information beyond LVEF and independently predicts postoperative LV dysfunction and adverse outcomes [49].

Some heterogeneity exists, however. Pandis et al. [48] reported that patients with preoperative LV–GLS below −20.5% experienced a greater postoperative decline in LVEF, while LV–GLS values below −17.9% predicted >10% reduction in LVEF and postoperative LVEF below 50%. Conversely, Song et al. [31] found no significant association between preoperative GLS and early postoperative LV dysfunction, possibly due to immediate postoperative hemodynamic instability and variability in patient populations. Overall, impaired LV–GLS appears to be a sensitive marker of adverse outcomes in PMR, although the precise cut-off values vary slightly depending on the imaging platform and analysis software.

**Figure 2:**
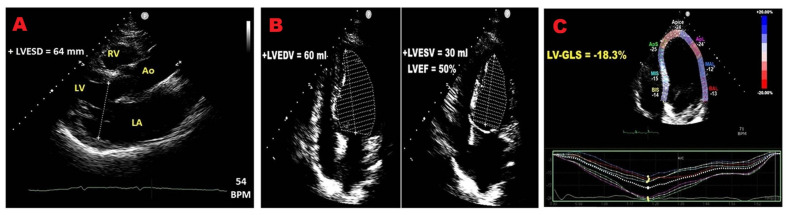
TTE and STE-derived LV indicators of adverse outcome in PMR individuals. (**A**) Enlarged LV end-systolic diameter. (**B**) Reduced LVEF. (**C**) Impaired LV–GLS. GLS, global longitudinal strain; LV, left ventricular; LVEF, left ventricular ejection fraction; LVEDV, left ventricular end-diastolic volume; LVESD, left ventricular end-systolic diameter; LVESV, left ventricular end-systolic volume; PMR, primary mitral regurgitation; STE, speckle tracking echocardiography; TTE, transthoracic echocardiography.

Figure 2 illustrates the main TTE and STE-derived indicators of poor prognosis in PMR individuals.

### 3.5. Left Atrial Size

Chronic volume overload in PMR leads to LA remodeling, which has been consistently linked to atrial fibrillation [50,51], mortality [52,53,54], and other adverse outcomes [50,52,53]. LA enlargement, assessed by diameter or indexed volume, thus represents a sensitive marker of disease severity and a useful predictor of prognosis [50,51,52,53,54].

### 3.6. Left Atrial Reservoir Strain

Left atrial strain (LASr) has emerged as a sensitive marker of atrial function and remodeling [55]. Several studies have shown that reduced LASr is independently associated with postoperative adverse outcomes, including mortality, cardiac events, LV dysfunction, and impaired functional capacity [47,56,57]. An LASr cut-off of ~21% has been reported to predict postoperative cardiac events [58]. Multicenter studies have further demonstrated that impaired LASr independently predicts long-term mortality and provides incremental prognostic information over LA volume [59]. Moreover, impaired LASr has been associated with adverse outcomes even in patients with moderate, asymptomatic MR, highlighting its potential role in refining early risk stratification [60].

Interestingly, LASr impairment may precede changes in LV strain. Cameli et al. [61] proposed that because of the thin atrial wall and chronic volume overload, the LA is particularly vulnerable, and LASr abnormalities may appear earlier than LV strain impairment. Future investigations should consider the absence of universal normal values across vendors and the technical challenges associated with thin atrial walls [62].

**Figure 3:**
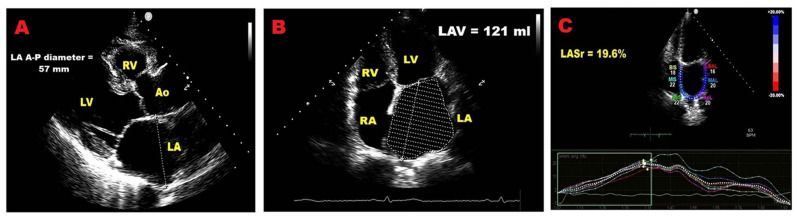
TTE and STE-derived LA indicators of adverse outcome in PMR individuals. (**A**) Enlarged LA diameter. (**B**) Increased LA volume. (**C**) Reduced LASr. A-P, antero-posterior; Ao, aorta; LA, left atrial; LASr, left atrial reservoir strain; LAV, left atrial volume; LV, left ventricular; PMR, primary mitral regurgitation; RA, right atrial; RV, right ventricular; STE, speckle tracking echocardiography; TTE, transthoracic echocardiography.

Representative examples of key transthoracic and speckle-tracking echocardiography-derived LA indicators, associated with adverse outcomes in PMR, are shown in Figure 3.

### 3.7. Pulmonary Hypertension

Pulmonary hypertension (PH) complicates approximately 20–30% of severe PMR cases [63,64]. It reflects retrograde transmission of LA pressure, with subsequent RV afterload increase and remodeling [65]. PH has been associated with higher perioperative mortality [66], long-term mortality [63,64,67,68,69], adverse events [68], reoperation [70], and persistent postoperative PH [71]. An international multicenter cohort showed that PH nearly doubles the risk of death and heart failure in degenerative MR due to flail leaflet [72]. Thus, sPAP assessment adds important incremental prognostic information.

### 3.8. Right Ventricular Size and Function

RV involvement signals advanced disease and portends poor outcomes. Dilatation (RVEDD >35 mm) predicts prolonged ICU stay [73], while reduced RV fractional area change or elevated myocardial performance index (≥0.50) correlates with in-hospital mortality and circulatory failure [74]. Impaired RV ejection fraction (≤35%) is linked to higher cardiovascular mortality [75]. Tissue Doppler-derived s′ <8.75 mm/s and TAPSE <17.5 mm predict postoperative LV dysfunction [76], highlighting the prognostic value of a comprehensive RV evaluation in PMR.

### 3.9. Right Ventricular Strain

Evidence regarding RV strain in PMR remains limited. Kislitsina et al. [47] reported that impaired RV free-wall longitudinal strain correlated with postoperative LV dysfunction, and the combination of LV–GLS, RV strain, and LASr improved prognostic accuracy; however, RV strain did not independently predict survival in multivariable Cox regression analysis. Further studies are required to clarify the role of RV strain in prognostic stratification of PMR patients.

### 3.10. Functional Tricuspid Regurgitation

Functional tricuspid regurgitation (FTR) occurs in 25–59% of PMR patients [77,78], with moderate-to-severe cases present in 8–45% [79,80]. Its severity correlates with worse survival [81,82,83], higher risk of heart failure [78,79], and frequent progression during follow-up [78]. Combined mitral and tricuspid intervention has therefore gained increasing acceptance, supported by evidence of improved outcomes [84]. Surgical correction is strongly advised in patients with severe concomitant FTR undergoing mitral surgery, and may be considered in progressive FTR with annular dilatation or right heart failure symptoms [9].

**Figure 4:**
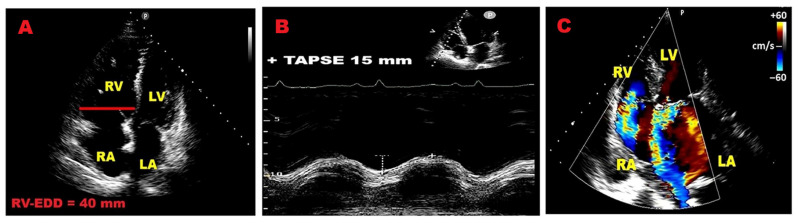
Relevant RV indicators of adverse outcome in PMR individuals. (**A**) Enlarged RV–EDD. (**B**) Reduced TAPSE. (**C**) Moderate-to-severe FTR. EDD, end-diastolic diameter; FTR, functional tricuspid regurgitation; LA, left atrial; LV, left ventricular; PMR, primary mitral regurgitation; RA, right atrial; RV, right ventricular; TAPSE, tricuspid annular plane systolic excursion.

The most relevant TTE-derived RV indicators associated with adverse outcomes in PMR are shown in Figure 4.

## 4. Prognostic Indicators Assessed Using Exercise Stress Echocardiography

Exercise stress echocardiography (ESE) allows dynamic evaluation of mitral regurgitant volume and pulmonary pressures during peak exertion and is particularly valuable when symptoms and resting MR severity are discordant [85,86]. It helps reveal latent symptoms in apparently asymptomatic patients and guides recommendations for physical activity. Despite its clinical utility and safety, registry data (e.g., VHD II survey) show ESE remains underused in asymptomatic individuals [1].

ESE clarifies the cardiac origin of exertional dyspnea and provides prognostic information in MR [87]. In patients with PMR undergoing ESE before mitral surgery, lower achieved metabolic equivalents predict poorer long-term outcomes [88]. The technique also detects subclinical ventricular dysfunction through abnormal echocardiographic parameters or absent contractile reserve, and guidelines recommend its use when discrepancies exist between MR severity, LV function, and symptoms [8,9].

Several exercise-derived indices carry prognostic value (Supplementary Table S1). Exercise LVEF < 68% [89], LV end-systolic volume index ≥ 25 mL/m^2^ [89], and LV–GLS normalized for LVESD worse than −5.7%/cm [90] predict postoperative LV dysfunction. Exercise pulmonary hypertension (sPAP > 60 mmHg), reduced TAPSE (<26 mm), elevated E/e′ ratio, larger EROA, and positive stress responses are linked to adverse postoperative outcomes [91,92]. Contractile reserve of LVEF (Δ ≥ 4%) and LV–GLS (Δ ≥ 1.9%) independently predict postoperative LV dysfunction [93].

ESE identifies dynamic MR—marked increases in regurgitation during exertion—which occurs in up to one-third of patients with at least moderate MR at rest [94,95]. Dynamic MR often coincides with exercise-induced pulmonary hypertension; sPAP >60 mmHg predicts symptom onset [96,97].

Detection of latent LV dysfunction is another key application. Limited contractile reserve, especially blunted LV–GLS augmentation (<2%) during exercise, identifies patients prone to early postoperative decompensation [90]. GLS often declines before LVEF, marking early myocardial dysfunction.

Diastolic stress echocardiography adds complementary insight by identifying elevated LV filling pressures during exertion. In healthy individuals, mitral inflow and annular velocities rise proportionally, maintaining stable E/e′ ratios [98]. In contrast, patients with diastolic dysfunction show increased E/e′ and/or sPAP correlating with invasive measures of elevated filling pressures [99]. Abnormal findings include septal E/e′ > 15, average E/e′ > 14, TRV >2.8 m/s, and reduced baseline e′ velocities [100]. Normal values correspond to septal E/e′ < 10 and TR velocity < 2.8 m/s at rest and during exercise.

Exercise-induced MR progression (ΔEROA ≥ 10 mm^2^, ΔRV ≥ 10 mL) [95], pulmonary hypertension (sPAP ≥ 60 mmHg) [96], and absent LV contractile reserve [94,101] are strong predictors of adverse outcomes. Emerging evidence links exercise RV dysfunction (TAPSE ≤ 18 mm) with increased risk [102]. Recent multicenter and meta-analytic studies confirm ESE’s prognostic power in MR, enhancing risk stratification in degenerative and asymptomatic cases [103,104,105].

## 5. Critical Appraisal of Established Echocardiographic Prognostic Markers in Primary Mitral Regurgitation

Transthoracic echocardiography remains the clinical standard for evaluating PMR, yet the prognostic weight of its established markers warrants nuanced appraisal. Traditional indices such as LVESD and LVEF are robust, reproducible, and validated in large cohorts, forming the backbone of current surgical thresholds. However, their predictive accuracy is limited by the late onset of abnormalities: both LVESD ≥ 40 mm and LVEF ≤ 60% often reflect irreversible remodeling, potentially missing the optimal timing for intervention. Left atrial size is a sensitive marker of chronic volume overload and correlates with atrial fibrillation and mortality, but its specificity is reduced by confounding conditions such as hypertension or diastolic dysfunction. Pulmonary hypertension provides incremental prognostic information, yet resting measurements fluctuate with loading conditions, and the prognostic utility of exercise-induced pulmonary pressures remains incompletely defined. Assessment of right ventricular size and function highlights advanced disease and correlates with perioperative risk, but technical challenges, geometric assumptions, and load dependence reduce reliability. Functional tricuspid regurgitation, although recognized as a marker of adverse outcomes, often progresses dynamically and lacks standardized quantification methods, complicating surgical decision-making.

Speckle tracking echocardiography (STE) offers earlier detection of subclinical dysfunction through strain analysis, particularly global longitudinal strain and atrial strain. Nonetheless, STE is hampered by inter-vendor variability, image quality dependence, and a lack of universally accepted thresholds. Load dependence and technical challenges in measuring atrial strain in thin-walled structures further limit generalizability.

Exercise stress echocardiography adds dynamic information, unmasking latent symptoms, contractile reserve, and exercise-induced pulmonary hypertension. Despite this, ESE is underutilized in clinical practice and suffers from several limitations. Outcomes are strongly dependent on patient effort and exercise capacity, potentially confounding interpretation. Moreover, there is no consensus on cut-offs for stress-derived parameters such as stress LVEF, ΔGLS, or peak sPAP, and inter-laboratory variability in protocols undermines reproducibility. Hemodynamic changes during exercise may be transient and difficult to capture consistently.

Table 2 summarizes traditional transthoracic echocardiographic parameters, speckle tracking echocardiography, and exercise stress echocardiography, highlighting their incremental value for risk stratification as well as their methodological limitations and unmet research needs.

## 6. Contributive Role of Cardiac MRI and PET/MR in Prognostic Stratification of Primary Mitral Regurgitation

Beyond echocardiography, advanced multimodality imaging techniques have emerged as valuable tools for assessing myocardial deformation and tissue remodeling in patients with PMR. Cardiac magnetic resonance (CMR) provides highly reproducible strain quantification through feature tracking and myocardial tagging, with incremental prognostic value over conventional indices. Romano et al. [106] demonstrated that CMR-derived GLS identifies early subclinical dysfunction and predicts adverse postoperative outcomes in PMR with preserved ejection fraction, complementing speckle tracking echocardiography. In parallel, tissue characterization with late gadolinium enhancement (LGE) and T1 mapping has proven effective in detecting both replacement and interstitial fibrosis. The Mitral FINDER study [107] showed that elevated extracellular volume (ECV) correlates with impaired strain, reduced exercise capacity, and biomarker elevation, even in asymptomatic patients. Similarly, Badau Riebel and Agoston-Coldea [108] reported that diffuse fibrosis, as assessed by ECV, is independently associated with adverse outcomes, highlighting the prognostic utility of CMR tissue characterization. Comprehensive reviews have further emphasized the role of CMR in capturing myocardial fibrosis and strain abnormalities that often precede overt functional decline [109]. Beyond CMR, hybrid positron emission tomography/magnetic resonance (PET/MR) has been explored in degenerative mitral valve disease: Miller et al. [110] reported increased FDG uptake in mitral valve prolapse, suggesting that metabolic imaging may provide complementary insights into inflammatory or ischemic substrates influencing strain. Collectively, these studies support the contributive role of CMR and hybrid imaging in refining risk stratification of PMR by integrating myocardial strain assessment with fibrosis characterization.

## 7. Laboratory Prognostic Indicators

Natriuretic peptides, particularly B-type natriuretic peptide (BNP) and its precursor fragments, have emerged as important biomarkers in mitral regurgitation. Plasma BNP levels rise in proportion to MR severity and are consistently higher in symptomatic compared with asymptomatic patients, even when LVEF remains preserved [111].

In organic MR, BNP activation primarily reflects the hemodynamic burden imposed on the ventricles and atria rather than the regurgitant volume itself. Elevated BNP levels have been shown to predict adverse outcomes under conservative management, regardless of MR grade [112]. This highlights BNP as a marker not only of disease severity but also of poor prognosis in patients managed medically. As such, BNP assessment is increasingly regarded as a valuable adjunct for risk stratification in PMR.

In asymptomatic patients with significant PMR and preserved LVEF undergoing MV surgery, the combination of BNP levels and LV–GLS has demonstrated complementary prognostic value. Together, these measures provide a synergistic framework for risk stratification beyond traditional markers [85].

## 8. Surgical or Percutaneous Treatment of PMR

Management of severe primary mitral regurgitation is traditionally guided by LV dimensions and ejection fraction. However, recent evidence suggests that LV–GLS provides incremental prognostic information in patients undergoing surgical repair [45,54].

To further refine risk stratification, the Mitral Regurgitation International Database (MIDA) score was developed to predict all-cause mortality in patients with severe PMR due to flail leaflet, whether managed surgically or medically [28]. The score incorporates variables such as symptoms, LVEF ≤ 60%, LVESD ≥ 40 mm, AF, right ventricular systolic pressure ≥ 50 mmHg, LA diameter ≥ 55 mm, and age ≥ 65 years. Of note, LVESD ≥ 40 mm and LA diameter ≥ 55 mm are now recognized thresholds in current guidelines.

Surgery is the treatment of choice for patients with symptomatic severe PMR and acceptable operative risk, as determined by the Heart Team. Independent of symptomatic status, triggers for intervention include LVEF ≤ 60%, LVESD ≥ 40 mm [28], LA diameter ≥ 55 mm or volume ≥ 60 mL/m^2^ [113], systolic pulmonary artery pressure (sPAP) > 50 mmHg [72], and AF [114,115]. In asymptomatic patients without these risk factors, watchful waiting remains appropriate, ideally within a Heart Valve Clinic setting [116].

When surgery is indicated, mitral valve repair is generally preferred over replacement, provided durable results are achievable, as it is associated with superior long-term survival [117,118]. Segmental prolapse due to degenerative disease can typically be repaired with excellent durability and low reoperation rates [118,119]. In contrast, rheumatic lesions, extensive prolapse, leaflet calcification, or severe annular calcification present greater challenges and may reduce reparability [120,121]. For complex lesions, surgery should be performed in high-volume repair centers with demonstrated expertise and outcomes. If repair is not feasible, valve replacement with preservation of the subvalvular apparatus is the recommended alternative.

In patients with contraindications to surgery or high operative risk, transcatheter approaches represent a safe alternative. Transcatheter mitral valve implantation has been demonstrated to be feasible and effective in high-risk cohorts [122,123]. Among these techniques, transcatheter edge-to-edge repair (TEER) is currently the most widely validated, with additional transcatheter strategies under evaluation in smaller studies [124,125].

Indications for TEER in PMR are currently limited to patients with severe symptoms, high or prohibitive surgical risk, and suitable valve anatomy [126]. For asymptomatic individuals with severe PMR and preserved LVEF (>60%), close follow-up every six months with echocardiography is advised, preferably in a Heart Valve Center [127]. Additional tools, such as BNP measurement, ESE, Holter monitoring, and cardiac magnetic resonance (CMR) imaging, may provide complementary value in guiding risk stratification and timing of intervention [24].

## 9. Innovative Anthropometric Prognostic Indicators of PMR Severity

### 9.1. Modified Haller Index

**Figure 5:**
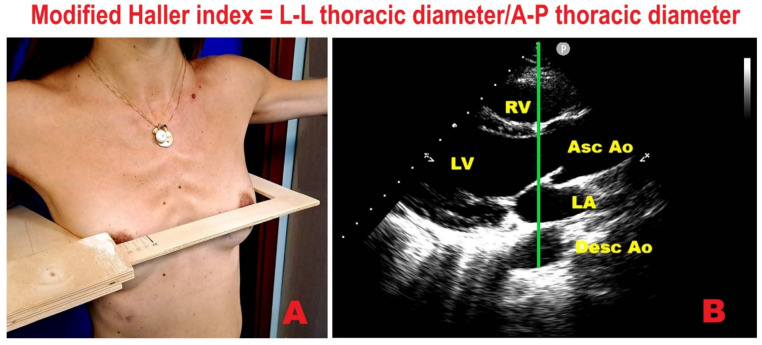
Modified Haller Index. (**A**) Latero-lateral external thoracic diameter measured with a rigid ruler and level at the distal third of the sternum. (**B**) Antero-posterior internal thoracic diameter obtained on transthoracic echocardiography in the parasternal long-axis view, measured as the distance from the true apex of the ultrasound sector to the posterior wall of the descending aorta immediately behind the left atrium. Ao, aorta; Asc, ascending; Desc, descending; LA, left atrium; LV, left ventricle; RV, right ventricle.

The Modified Haller Index (MHI) is a simple, noninvasive anthropometric measure of chest wall conformation that avoids ionizing radiation while yielding information comparable to radiographic indices [15]. It is computed as the ratio between the latero-lateral external thoracic diameter, measured with a rigid ruler and level, and the antero-posterior (A-P) internal thoracic diameter, acquired during standard transthoracic echocardiography from the parasternal long-axis view as the distance between the true apex of the sector and the posterior wall of the descending aorta visualized behind the left atrium (Figure 5).

Over recent years, we have examined how chest wall configuration may influence myocardial mechanics, MR grading, and ESE findings in individuals with PMR secondary to MVP. We hypothesized that a concave thorax, defined echocardiographically as MHI > 2.5 or A-P diameter ≤ 13.5 cm [21], might exert external compressive effects on cardiac chambers and the mitral annulus, thereby potentially affecting two-dimensional speckle-tracking echocardiography (2D–STE), conventional echocardiography, and exercise hemodynamics. In a prospective study of healthy MVP subjects without severe MR compared with matched controls, MVP participants exhibited a narrower A-P thoracic diameter consistent with a tendency toward mild-to-moderate chest deformity and showed more pronounced impairment of LV–GLS and global circumferential strain (GCS), particularly in basal segments, despite preserved biventricular systolic function on conventional echocardiography [18]. When stratified by MHI, MVP individuals with MHI > 2.5 demonstrated lower magnitudes of both longitudinal and circumferential strain and more abnormal LV twist mechanics than those with MHI ≤ 2.5, with these differences being most evident at the basal level. MVP participants with MHI > 2.5 were predominantly women with small body surface area and small cardiac chambers, more often had nonclassic MVP without myxomatous degeneration and late-systolic mild or mild-to-moderate MR, and more frequently exhibited isolated ventricular premature beats and nonspecific ST–T abnormalities on resting ECG. Across MVP and control groups, MHI correlated linearly and strongly with LV–GLS and LV–GCS.

**Figure 6:**
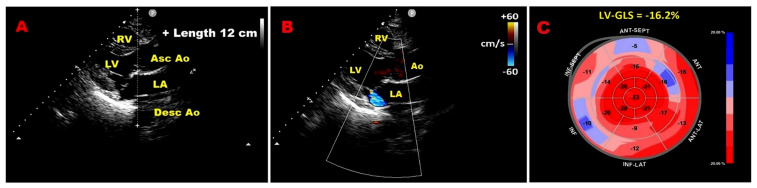
Example of MVP with concave chest wall conformation (MHI > 2.5 or A-P diameter ≤ 13.5 cm). (**A**) Narrow A-P thoracic diameter (≤13.5 cm) measured from the parasternal long-axis view. (**B**) Parasternal long-axis view showing mild MR on color Doppler. (**C**) 2D–STE bull’s eye plot revealing mild-to-moderate impairment of LV–GLS, most marked in basal segments. 2D, two-dimensional; Ao, aorta; A-P, antero-posterior; Asc, ascending; Desc, descending; GLS, global longitudinal strain; LA, left atrium; LV, left ventricle; MHI, Modified Haller Index; MVP, mitral valve prolapse; MR, mitral regurgitation; RV, right ventricle; STE, speckle tracking echocardiography.

Figure 6 illustrates an example of an MVP individual with concave-shaped chest wall conformation, mild MR, and mild-to-moderate impairment of LV–GLS, more enhancethe level of basal myocardial segments.

These findings differ from earlier reports that attributed reduced basal strain in MVP primarily to annular dilation [128,129,130]. In our cohort, MVP with concave chest morphology had shorter mitral annular A-P diameters yet worse global and regional strain, suggesting an association with thoracic mechanics rather than proving intrinsic myocardial disease. The mechanistic interpretation is that strain abnormalities typically precede overt contractile impairment, especially for GLS, with concomitant reductions across longitudinal, circumferential, and radial components usually reflecting more advanced systolic dysfunction and reduced ejection fraction [131,132,133]. In our MVP cohort, LVEF and RVEF were preserved while all strain measures were attenuated, which argues against, but does not exclude, a purely intrinsic myocardial explanation. The preserved apex-to-base deformation gradient, a pattern maintained from infancy to adulthood [134] and usually altered in cardiomyopathies and ischemia [135], also suggests that primary myopathic dysfunction is unlikely. Instead, our observations align with prior work indicating smaller biventricular chamber dimensions and lower strain in individuals with concave chest walls [136,137,138]. As demonstrated by CMR [138], relatively higher mid-apical strain in concave chest morphology may represent compensation to maintain stroke volume in the presence of possible basal sternal constraint. The graded relationship between MHI and strain impairment appears consistent with previous data in healthy subjects with pectus excavatum [16,17], indicating an extrinsic or geometric mechanism rather than a direct causal effect. In MVP with MHI > 2.5, increased apical rotation with blunted basal rotation could reflect a rocking motion around a fulcrum near the shortest A-P chest diameter, a kinematic pattern that may confound speckle-tracking algorithms and simulate strain reduction despite preserved intrinsic contractility.

### 9.2. Relationship Between Chest Wall Conformation and MAD Distance in PMR with MVP

The association of PMR with sudden cardiac death and ventricular arrhythmias remains debated [139,140]. Mitral annular disjunction (MAD), defined as atrial displacement of the mitral hinge line away from the ventricular myocardium, has been linked to ventricular arrhythmias and increased arrhythmic risk in MVP, often in the absence of severe MR [139,141,142]. Given the frequent coexistence of MVP with thoracic skeletal anomalies and the observed enrichment of MAD in MVP populations [143,144,145,146,147,148,149,150], we hypothesized that MAD would be more prevalent in individuals with narrow A-P thoraces and concave chest morphology. In a prospective monocentric study of MVP with and without MAD, patients with MAD had significantly smaller A-P thoracic diameters on TTE, smaller cardiac chambers, a higher prevalence of classic (floppy) MVP, and more impaired LV–GLS and LV–GCS despite similar LVEF when compared with those without MAD [151]. Multivariable analyses identified A-P thoracic diameter, classic MVP, and end-systolic mitral annular A-P diameter as independent predictors of MAD. MAD distance correlated strongly with MHI and A-P diameter but not with latero-lateral diameter. Among MAD-positive patients, MHI correlated inversely with both LV–GLS and LV–GCS, whereas this relationship was not evident in MAD-negative subjects. These findings suggest that a narrow A-P chest is associated with MAD and is a major determinant of impaired myocardial deformation in MAD-positive MVP, without commensurate differences in resting MR severity between groups that both exhibited moderate MR.

**Figure 7:**
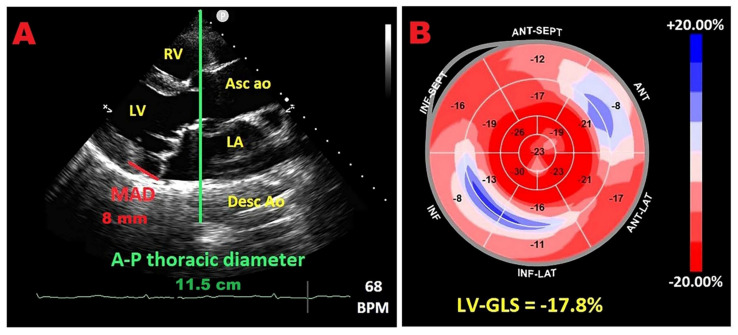
Representative example of an MVP individual with MAD, narrow A-P thoracic diameter, and mild deterioration of LV–GLS with “apical sparing” pattern. (**A**) Parasternal long-axis view showing MVP, MAD, and reduced A-P diameter (red line indicates MAD distance; green line indicates A-P thoracic diameter). (**B**) 2D–STE bull’s eye plot depicting mild LV–GLS reduction with predominant basal involvement and relative apical sparing. Ao, aorta; A-P, antero-posterior; Asc, ascending; Desc, descending; GLS, global longitudinal strain; LV, left ventricle; MAD, mitral annular disjunction; MVP, mitral valve prolapse; RV, right ventricle.

Among the MVP patients included in the present study, those with MAD exhibited greater impairment in myocardial strain parameters, especially at the basal level, in both longitudinal and circumferential directions (Figure 7).

The basal pattern of strain attenuation in MAD may further reflect the influence of anterior chest wall mechanics in limiting basal motion and possibly contributing to regional dyssynchrony [17,18]. These features can also be affected by acoustic-window quality and body-habitus artifacts, and may therefore be accentuated or misinterpreted as intrinsic basal hypokinesia even by experienced readers. The strong inverse associations between MHI and both LV–GLS and LV–GCS in MAD patients, concordant with our prior studies across different populations [16,17,18,20], support the possibility of a mechanical contribution in addition to any underlying myocardial substrate. This interpretation contrasts with reports ascribing basal strain impairment in MAD to annular dilation and intrinsic LV remodeling using 2D–STE [128], 3D transesophageal echocardiography [152], and CMR [153]. In our MAD cohort, approximately one-third had MAD distance ≥ 8.5 mm, three-quarters had isolated ventricular premature beats on resting ECG, and more than four-fifths had classic MVP with enlarged annular diameters and paradoxical systolic annular unsaddling. These features support the concept that disjunction may amplify leaflet stress and could contribute to myxomatous degeneration [154,155,156,157], while excessive leaflet mobility and paradoxical annular dynamics might promote inferobasal and papillary-muscle stretch, hypertrophy, fibrosis, and scar formation, with arrhythmogenic consequences [139,158,159,160,161]. The inverse relationship between MAD distance and A-P diameter suggests that chronic anterior chest mechanics may contribute to or coexist with both MVP and MAD. As others have proposed [162,163], a concave chest wall or pectus excavatum, MVP, and MAD may cluster as a syndromic trait in some individuals.

### 9.3. Potential Link Between Concave Chest Morphology and a “Benign MAD Phenotype”

In early 2025, we reported an MVP case with significant MAD and moderate MR who underwent a comprehensive imaging work-up, including TTE, transesophageal echocardiography, CMR, coronary CT angiography, and ESE [164]. The patient had a concave chest configuration (MHI > 2.5; A-P diameter < 13.5 cm), hemodynamically non-significant MR on ESE, no late gadolinium enhancement on CMR, and a low arrhythmic burden. These observations led us to hypothesize that MHI might help identify a subset of MVP with MAD who carry a relatively favorable prognosis. In a subsequent series, MVP patients with MAD and MHI > 2.5 or A-P diameter < 13.5 cm exhibited a low prevalence of complex ventricular arrhythmias and favorable mid-term outcomes, supporting the concept of a “benign MAD phenotype” associated with concave chest morphology [165].

### 9.4. Influence of Chest Wall Conformation on ESE Results

To explore how chest wall anatomy influences ESE findings, we conducted a retrospective analysis of a consecutive cohort of 424 consecutive symptomatic patients with MVP and moderate MR who underwent ESE at our center between February 2014 and February 2021 [22]. The primary reason for testing was a mismatch between reported symptoms (dyspnea at rest or during exertion, chest pain, and/or palpitations) and resting echocardiographic evidence consistent with only moderate MR. MHI was measured noninvasively in all patients. At peak exercise, positive ESE was observed in 9.8% and exercise-induced severe MR (ΔEROA ≥ 0.13 cm^2^) in 13.2%. During a mean follow-up of 3.2 ± 1.7 years, 75 cardiovascular events occurred, including 55 hospitalizations (heart failure, acute coronary syndromes, or arrhythmias) and 20 mitral valve interventions. Univariate Cox analysis identified several clinical, ECG, and Doppler predictors of MACE, with MHI showing a significant inverse correlation with outcomes. Multivariate analysis confirmed that age, diabetes, peak exercise E/e′ ratio, and peak exercise EROA were independently associated with adverse events, while MHI and beta-blocker therapy were protective. ROC analysis indicated an MHI ≥ 2.7 predicted event-free survival with high accuracy (AUC = 0.98). Unlike previous studies [93,99,100,101,112,166,167], this investigation assessed both conventional ESE prognostic markers and a novel anthropometric parameter (MHI), measured using a non-radiological method [15]. Patients with concave chest wall (MHI ≥ 2.7) had fewer MACE, consistent with the literature showing that age, diabetes, impaired stress echo indices, and lack of beta-blocker use predict worse outcomes [168,169]. Both univariate and multivariate models confirmed MHI > 2.5 as independently protective.

**Figure 8:**
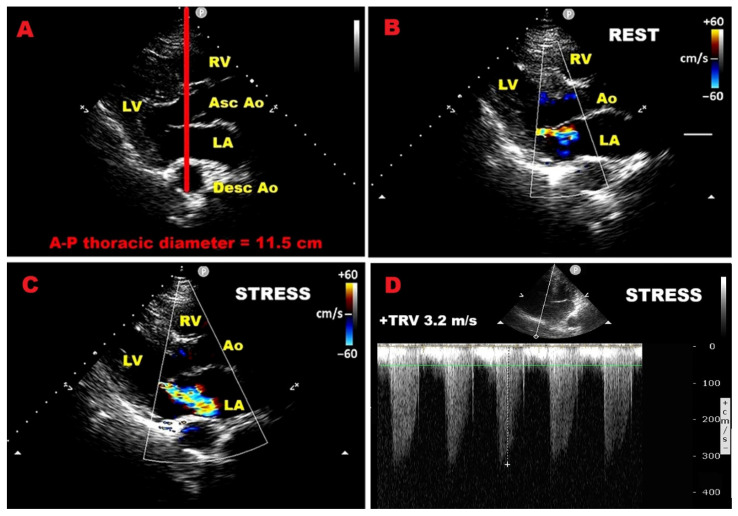
Dynamic MR assessment using ESE in symptomatic MVP with concave thorax (A-P diameter ≤ 13.5 cm) and resting mild-to-moderate MR. (**A**) A-P diameter measurement in the parasternal long-axis view as the distance between the true sector apex and the posterior wall of the descending aorta. (**B**) Parasternal long-axis view showing resting mild-to-moderate MR. (**C**) Parasternal long-axis view revealing peak-exercise moderate MR. (**D**) Continuous-wave Doppler-derived peak exercise TRV of 3.2 m/s (RV–RA gradient 42 mmHg), excluding exercise-induced severe pulmonary hypertension (sPAP < 60 mmHg). ESE, exercise stress echocardiography; MR, mitral regurgitation; MVP, mitral valve prolapse; PH, pulmonary hypertension; RA, right atrial; RV, right ventricular; sPAP, systolic pulmonary artery pressure; TRV, tricuspid regurgitation velocity.

Figure 8 reports an example of dynamic MR assessment using ESE performed in a symptomatic MVP individual with concave-shaped chest wall conformation (A-P thoracic diameter ≤ 13.5 cm) affected by resting mild-to-moderate MR. ESE revealed a mild increase in MR degree (estimated to be moderate at peak exercise), with no evidence of severe PH (sPAP < 60 mmHg).

The overall profile of this cohort helps explain the favorable outcomes. Most participants had well-controlled blood pressure and a modest burden of other cardiovascular risk factors, thereby limiting the additive mechanical load that hypertension imposes on LV pressure and regurgitant volume [170]. The majority exhibited mid–late systolic rather than holosystolic MR, the latter being more strongly associated with adverse prognosis [167]. MR severity may be overestimated on resting TTE in concave thoraces, especially when LA size is small, because single-frame measures such as vena contracta width and proximal isovelocity surface area can be influenced by jet eccentricity and suboptimal alignment, particularly in MVP with tunneling jets and spray effects within a small receiving chamber [171,172]. Purely mid–late systolic MR has lower regurgitant volume and milder hemodynamic consequences than holosystolic MR; in such cases, regurgitant volume is a more reliable reflection of severity than instantaneous EROA. Accordingly, clinical decisions should incorporate MR timing and consequences rather than single-frame jet parameters [171]. Finally, beta-blocker therapy was associated with a 64% reduction in cardiovascular events, an effect plausibly mediated by attenuating sympathetic activation in MVP [173,174].

### 9.5. Implications for Clinical Practice

**Figure 9:**
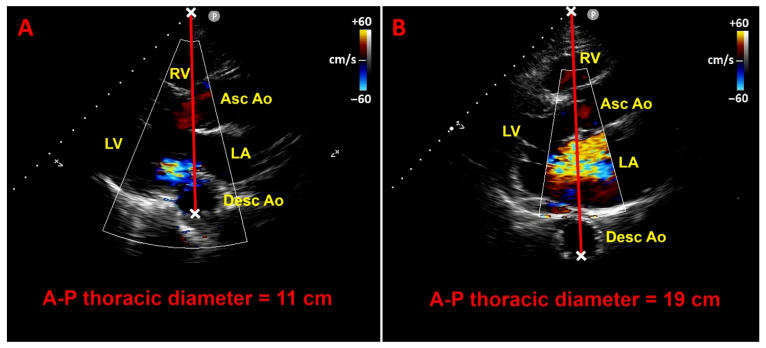
Parasternal long-axis view illustrating A-P thoracic assessment in two MVP patients. (**A**) MVP individuals with narrow A-P diameter (≤13.5 cm) and mild MR. (**B**) MVP individual with a more circular transverse thoracic section and moderate-to-severe MR. Red lines indicate the A-P sterno-vertebral distance. Ao, aorta; A-P, antero-posterior; Asc, ascending; Desc, descending; LA, left atrium; LV, left ventricle; MR, mitral regurgitation; MVP, mitral valve prolapse; RV, right ventricle.

In a middle-aged, otherwise healthy MVP individual—particularly a woman—with narrow A-P thorax (MHI > 2.5) and preserved LVEF, detection of regional (especially basal) and/or global strain impairment should prompt consideration of chest conformation artifacts on 2D–STE rather than automatic attribution to intrinsic myocardial dysfunction. A diffuse basal reduction or a global strain decrement does not respect coronary territories and may avert unnecessary testing for myocardial ischemia. Within MVP populations, a narrow A-P diameter (≤13.5 cm) confers a higher probability of detecting MAD on standard TTE, while a more circular transverse thorax (A-P diameter > 13.5 cm) lowers MAD likelihood but often correlates with older male sex, comorbidity, larger chambers (especially LA), and moderate-to-severe, potentially hemodynamically significant MR during exertion (Figure 9).

The inverse relationship between MHI and both LV–GLS and LV–GCS is most evident in narrow A-P chests, again indicating that reduced strain values may partly arise from chest-wall-related mechanics and tracking limitations rather than primary myocardial disease. Speckle-tracking algorithms assume that the motion of gray-scale kernels remains within the imaging plane throughout the cardiac cycle [175]. When external thoracic geometry displaces the heart, alters its motion pattern, or changes the insonation angle, out-of-plane motion can appear as in-plane displacement, leading to apparent strain reduction similar to tangential interrogation of a normally contracting wall [175]. Clinicians should therefore recognize that strain decrements may result from geometric, acoustic, or kinematic factors, not solely from altered myocardial mechanics. Among MVP patients, those with MAD typically have shorter A-P diameters and more impaired deformation indices despite normal LVEF on conventional echocardiography. The compressive and kinematic effects of a narrow A-P chest could induce global and regional motion patterns that confound tracking and simulate intrinsic dysfunction. Incorporating a quick chest-shape assessment into routine MVP evaluation can therefore refine the interpretation of 2D–STE findings.

These principles also inform the use of ESE in moderate PMR. In centers without external rulers and levels, the A-P diameter can be derived from parasternal long-axis TTE alone to flag concave morphology or pectus excavatum. In patients with MHI > 2.5, small LV and LA sizes out of proportion to apparent MR grade, and non-holosystolic MR, clinicians should weigh the risk of overestimating MR and, in some cases, defer ESE for MR hemodynamic assessment or ischemia testing, given the low medium-term event rates and excellent prognosis in such individuals [165,176]. In symptomatic moderate PMR undergoing ESE, chest conformation correlates with outcomes; concave thoraces show fewer MACE over medium-term follow-up. An MHI ≥ 2.7 may identify a subgroup with a particularly favorable prognosis in whom ESE may be unnecessary.

**Figure 10:**
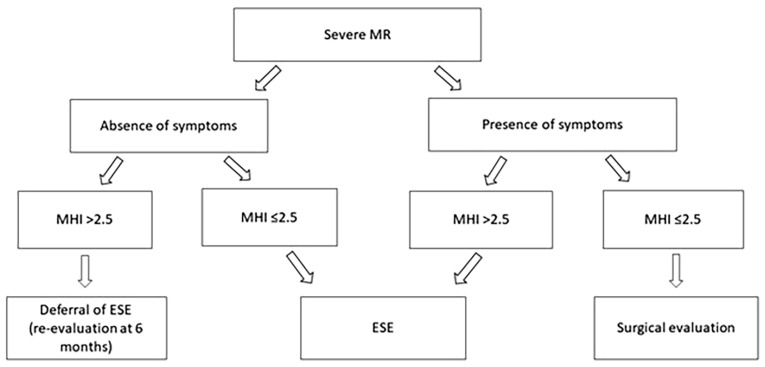
Suggested algorithm integrating the Modified Haller Index into the decision-making process for exercise stress echocardiography in patients with severe mitral regurgitation. ESE, exercise stress echocardiography; MHI, modified Haller index; MR, mitral regurgitation.

Figure 10 presents a schematic representation of a reasonable clinical pathway for the indication of ESE in patients with severe MR, incorporating the MHI as a morphological modifier. In individuals with severe asymptomatic MR and MHI > 2.5, usually corresponding to a narrow, concave thorax with small cardiac chambers, it is reasonable to defer ESE and schedule re-evaluation after approximately six months. In this subgroup, MR severity may be overestimated because of geometric and acoustic artifacts related to thoracic conformation, and medium-term prognosis is generally favorable. Conversely, in severe symptomatic MR with MHI ≤ 2.5, typically associated with a more circular thoracic configuration and genuinely hemodynamically significant MR, it is reasonable to perform ESE to confirm exercise-induced MR progression or pulmonary hypertension. When stress findings support a true severe MR, subsequent surgical evaluation should be considered. This algorithm underscores the value of incorporating chest wall assessment into the decision process, ensuring that ESE is selectively applied to patients most likely to derive diagnostic and prognostic benefit.

### 9.6. Limitations of Innovative Anthropometrics in Assessing PMR Severity

Although the MHI represents an intriguing and original approach to refining prognostic assessment in PMR, its evidence base remains exploratory, and its practical and prognostic validity requires cautious interpretation. Current data derive predominantly from single-center observational studies, many conducted by our own research group, which raises concerns about potential self-citation bias, external reproducibility, and publication bias. These observations should therefore be regarded as preliminary associations rather than definitive evidence of causality. Despite the consistent findings across our series, these results require confirmation by independent external investigators to ensure generalizability and mitigate the influence of institutional methodology or population characteristics. The available studies are limited by modest sample sizes, short follow-up, and a predominant focus on MVP rather than the broader PMR population, which limits generalizability.

From a methodological perspective, the original validation of the MHI demonstrated a strong correlation with the radiological Haller Index (r = 0.81, *p* < 0.001) and tight limits of agreement on Bland–Altman analysis (−0.51 to +0.37) [15], supporting its feasibility and inter-method reliability. However, formal inter- and intra-observer reproducibility testing across different echocardiographic operators and equipment has not yet been systematically performed. Measurement variability may arise from differences in parasternal imaging planes, respiratory phase, soft-tissue inclusion, and patient positioning, particularly when using transthoracic echocardiography to estimate the antero-posterior chest diameter. These potential sources of error should be explicitly acknowledged when interpreting MHI-related findings.

Furthermore, the reported correlations between chest wall conformation and myocardial strain parameters remain observational and cannot confirm a direct mechanical or pathophysiological link. These associations may in part reflect acoustic-window or body-habitus effects that influence speckle-tracking algorithms. Controversy persists as to whether MHI truly captures pathophysiologically relevant geometry or merely reflects imaging artifacts. Future research should address whether thoracic conformation independently affects MR dynamics, strain, and exercise hemodynamics beyond imaging limitations.

Open questions include the reproducibility of MHI measurement across platforms, its incremental prognostic value over conventional markers, and the potential thresholds defining clinically meaningful subgroups. Independent multicenter validation with standardized protocols and blinded reproducibility analysis is therefore essential before MHI can be considered for clinical decision-making or risk-stratification frameworks.

Overall, MHI should currently be viewed as a hypothesis-generating anthropometric descriptor—useful for research but not yet validated for clinical use—pending larger, prospective, and independently replicated studies to confirm its reproducibility, pathophysiological meaning, and prognostic value.

## 10. Conclusions

Systematic physical examination, including chest wall morphology assessment, may enhance evaluation of PMR severity. Noninvasive estimation of the MHI can help identify patients with narrow antero-posterior chest configuration and non-malignant mitral annular disjunction phenotypes, often showing moderate strain attenuation but favorable outcomes.

However, the clinical and prognostic utility of MHI remains exploratory. Current evidence is limited to single-center observational studies, without extensive assessment of inter- and intra-observer reproducibility or external validation. Measurement variability related to echocardiographic acquisition and body habitus further limits its applicability.

Future multicenter prospective studies should verify reproducibility, prognostic significance, and integration of MHI into multiparametric imaging models. Until such validation is available, MHI should be regarded as a hypothesis-generating anthropometric marker, useful for research and for highlighting the potential influence of chest wall conformation on myocardial deformation in PMR.

## Figures and Tables

**Table 1:** Principal qualitative, semiquantitative, quantitative, and structural indicators of PMR severity. CW, continuous; wave; EROA, effective regurgitant orifice area; LA, left atrium/left atrial; LVESD, left ventricular end-systolic diameter; MV, mitral valve; PISA, proximal isovelocity surface area; PMR, primary mitral regurgitation; TVI, time–velocity integral.

Category	Criteria
**Qualitative**	MV morphology (flail leaflet, papillary rupture, severe retraction, perforation); Large central jet (>50% LA) or eccentric jet; Large systolic flow convergence; Dense holosystolic CW Doppler jet
**Semiquantitative**	Vena contracta ≥ 7 mm; Systolic pulmonary vein flow reversal; Dominant E-wave (>1.2 m/s); TVI mitral/TVI aortic > 1.4
**Quantitative**	PISA radius ≥ 1 cm; EROA ≥ 40 mm^2^; Regurgitant volume ≥ 60 mL/beat; Regurgitant fraction ≥ 50%
**Structural**	LVESD ≥ 40 mm; LA diameter ≥ 55 mm or volume ≥ 60 mL/m^2^

**Table 2:** Prognostic value, limitations, and future perspectives of echocardiographic markers in primary mitral regurgitation. ΔGLS = change in global longitudinal strain (usually during stress compared with rest); FTR, functional tricuspid regurgitation; GLS, global longitudinal strain; LA, left atrial; LASr, left atrial reservoir strain; LV, left ventricular; LVEF, left ventricular ejection fraction; LVESD, left ventricular end-systolic diameter; MR, mitral regurgitation; sPAP, systolic pulmonary artery pressure; PH, pulmonary hypertension; PMR, primary mitral regurgitation; RV, right ventricular; TTE, transthoracic echocardiography.

Modality/Marker	Strengths	Limitations	Current Gaps/Future Needs
Traditional TTE indices (LVESD, LVEF, LA size, PH, RV indices, FTR)	Widely available; guideline-endorsed thresholds; strong prognostic validation in large cohorts; reproducible LV diameters.	Reflect late disease; load dependent; interobserver variability; confounded by comorbidities (e.g., hypertension, diastolic dysfunction); limited sensitivity for early dysfunction.	Refined cut-offs for earlier intervention; multiparametric risk models; standardized RV/FTR quantification.
Speckle tracking echocardiography (GLS, LASr, RV strain)	Sensitive to subclinical dysfunction; incremental prognostic value over LVEF; detects earlier atrial/ventricular remodeling.	Image quality dependent; vendor/software variability; lack of universal reference values; load dependent; atrial strain limited by thin wall.	Harmonization across vendors; multicenter validation; integration into surgical decision algorithms.
Exercise stress echocardiography	Provides dynamic assessment of MR severity, contractile reserve, and exercise-induced PH; unmasks latent symptoms; predicts outcomes beyond resting echo.	Underutilized; dependent on patient effort; no consensus on stress cut-offs (e.g., stress LVEF, ΔGLS, sPAP); variability in protocols; transient hemodynamics difficult to capture.	Standardized protocols and thresholds; broader implementation in asymptomatic PMR; multicenter trials linking ESE metrics to outcomes.

## Data Availability

Data extracted from included studies will be made publicly available on Zenodo (https://zenodo.org, accessed on 8 September 2025).

## Supplementary Materials

The following supporting information can be downloaded at: https://www.mdpi.com/article/10.3390/jcm14238297/s1, Table S1: Prognostic Indicators Assessed Using Exercise Stress Echocardiography.
