# Supplementary material for: Does Preliminary Chest Shape Assessment Improve the Prognostic Risk Stratification of Symptomatic Individuals with Primary Mitral Regurgitation? A Narrative Review of Traditional and Innovative Prognostic Indicators
Source: J Clin Med. 2025 Nov 22;14(23):8297. doi: 10.3390/jcm14238297 (PMC12693287; doi:10.3390/jcm14238297)
Supplement: Supplementary file 1 [file jcm-14-08297-s001.zip › jcm-3851487-supplementary.pdf]

Table S1. Prognostic Indicators Assessed Using Exercise Stress Echocardiography.

Category	Prognostic Indicator	Threshold/Definition	Clinical Implication
**LV systolic function**	Exercise LVEF	< 68%	Predicts postoperative LV dysfunction
	LVESVi	≥ 25 mL/m²	Predicts postoperative LV dysfunction
	LV–GLS normalized for LVESD	Worse than –5.7%/cm	Predicts postoperative LV dysfunction
	Contractile reserve (LVEF)	Δ ≥ 4%	Protective; absence predicts adverse outcomes
	Contractile reserve (LV–GLS)	Δ ≥ 1.9%	Protective; absence predicts adverse outcomes
	Blunted GLS increase	< 2% during exercise	Identifies early subclinical dysfunction
**Mitral regurgitation dynamics**	Increase in EROA	Δ ≥ 10 mm²	Predicts adverse CV outcomes
	Increase in regurgitant volume	Δ ≥ 10 mL	Predicts adverse CV outcomes
	Dynamic MR	MR worsens to severe during exercise	Linked with worse prognosis
**Pulmonary pressures**	Exercise sPAP	> 60 mmHg	Predicts symptom onset and adverse outcomes
**Right ventricular function**	Exercise TAPSE	≤ 18 mm (high risk)	Associated with poor prognosis and CV events
**Diastolic stress echo**	Septal E/e′	> 15 (abnormal)	Indicates elevated filling pressures
	Average E/e′	> 14 (abnormal)	Indicates elevated filling pressures
	TRV	> 2.8 m/s	Indicates exercise-induced PH
	Normal response	Septal E/e′ < 10 and TRV < 2.8 m/s	Favorable prognosis
